# PML Degradation: Multiple Ways to Eliminate PML

**DOI:** 10.3389/fonc.2013.00060

**Published:** 2013-03-22

**Authors:** Andrea Rabellino, Pier Paolo Scaglioni

**Affiliations:** ^1^Division of Hematology and Oncology, Department of Medicine, Simmons Comprehensive Cancer Center, University of Texas Southwestern Medical CenterDallas, TX, USA

**Keywords:** PML, tumor suppressor, ubiquitination, degradation, sumoylation

## Abstract

The promyelocytic leukemia tumor suppressor gene (PML) critically regulates several cellular functions that oppose tumorigenesis such as oncogene-induced senescence, apoptosis, the response to DNA damage and to viral infections. PML deficiency occurs commonly in a broad spectrum of human cancers through mechanisms that involve its aberrant ubiquitination and degradation. Furthermore, several viruses encode viral proteins that promote viral replication through degradation of PML. These observations suggest that restoration of PML should lead to potent antitumor effects or antiviral responses. In this review we will summarize the mechanisms involved in PML degradation with the intent to highlight novel therapeutic strategies to trigger PML restoration.

## Introduction

The promyelocytic leukemia tumor suppressor gene (PML) was initially identified as a component of the PML-Retinoic Acid Receptor Alpha (RARA) oncoprotein as a result of the chromosomal translocation *t*(15;17) of acute promyelocytic leukemia (APL) (de The et al., 1990; Goddard et al., 1991; Kakizuka et al., 1991; Pandolfi et al., 1991).

The human *PML* gene is located on chromosome 15, and consists of nine exons that generate several alternative spliced transcripts. As a result, all of the PML isoforms contain a conserved N-terminal region consisting of the RING, B-boxes, coiled-coil (RBCC) motifs, and the SUMO Binding Domain (SBD), and they all differ in the central and C-terminal regions (Fagioli et al., 1992; Jensen et al., 2001).

Promyelocytic leukemia tumor suppressor gene concentrates in distinct subnuclear structures known as PML-Nuclear Bodies (PML-NB), also called PML Oncogenic Domains (POD), Nuclear Domain 10 (ND10), or Kremer bodies (Bernardi and Pandolfi, 2007). PML-NBs are discrete highly dynamic nuclear foci with an average diameter of 0.2–1 μm. Typically, 5–30 PML-NBs are present in most mammalian cells nuclei, depending on the cell type and the cell cycle phase, while PML-NBs are disrupted and dispersed in microspeckles in the leukemic blasts of APL patients (Daniel et al., 1993; Dyck et al., 1994).

Promyelocytic leukemia tumor suppressor gene-nuclear bodies have been implicated in gene transcription (both activation and repression), apoptosis and cellular senescence, tumor suppression, viral pathogenicity, and DNA repair (Seeler and Dejean, 1999; Zhong et al., 2000a; Regad and Chelbi-Alix, 2001; Dellaire and Bazett-Jones, 2004; Bernardi and Pandolfi, 2007).

*Pml* null cells are devoid of PML-NBs: this observation led to the conclusion that PML is the essential component of PML-NBs (Wang et al., 1998a). Consistently, the functional properties of PML and PML-NBs overlap. For instance, PML critically regulates multiple tumor suppressive pathways such as oncogene-induced senescence (OIS) and apoptosis (Wang et al., 1998a,b; Ferbeyre et al., 2000; Pearson et al., 2000; Bernardi and Pandolfi, 2007; Bernardi et al., 2008). Moreover, *Pml* inactivation in mice leads to cancer susceptibility in several organs (Wang et al., 1998a; Rego et al., 2001; Scaglioni et al., 2006; Trotman et al., 2006). In humans, PML deficiency occurs commonly in a broad spectrum of human cancers including lung, prostate and breast carcinoma, lymphomas, CNS tumors, and germ cell tumors, through a mechanism that involves aberrant ubiquitin-mediated degradation (Koken et al., 1995; Gurrieri et al., 2004a; Scaglioni et al., 2006).

Due to the pivotal role of PML in tumor suppression and other important cellular functions, the understanding of the molecular mechanisms involved in its degradation have been the subject of intense investigation. This review will focus on the mechanisms involved in this process and will discuss their functional significance.

## PML Functions

**Figure 1:**
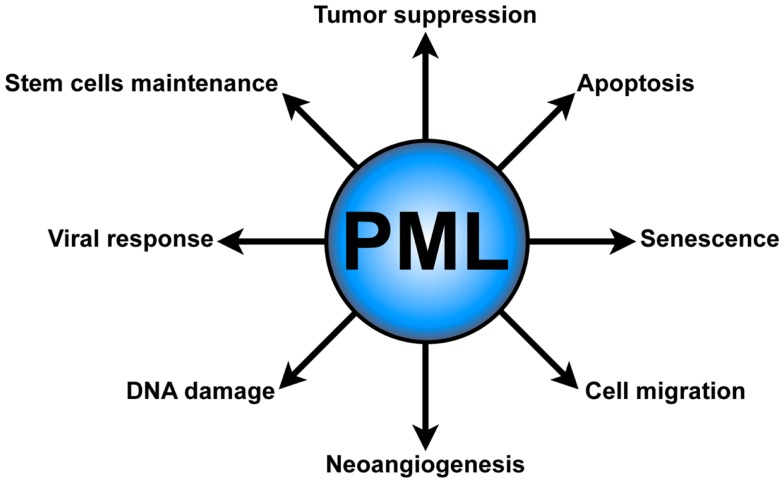
**PML functions**. PML plays several critical cellular functions, such as apoptosis and senescence, neoangiogenesis, cell migration, DNA damage response, antiviral defense, and hematopoietic stem cell maintenance.

Several lines of evidence underscore the role of PML in tumor suppression (Wang et al., 1998a; Rego et al., 2001; Scaglioni et al., 2006; Trotman et al., 2006), senescence, and apoptosis (Wang et al., 1998b; Ferbeyre et al., 2000; Guo et al., 2000; Pearson et al., 2000; Bischof et al., 2002; Scaglioni et al., 2012). Moreover, PML has been implicated in other important cellular functions, such as neoangiogenesis (Bernardi et al., 2006), cell migration (Reineke et al., 2010), the DNA damage response (Dellaire and Bazett-Jones, 2004), antiviral defense (Geoffroy and Chelbi-Alix, 2011), and most recently in the regulation of hematopoietic stem cells (HSCs) maintenance (Ito et al., 2012) (Figure 1).

### Cellular senescence

Replicative senescence is the phenomenon by which cells undergo an irreversible cell cycle arrest, loosing the ability to divide and proliferate. Several stresses have been implicated in the induction of senescence, including oxidative damage, telomerase dysfunction, DNA damage, and aberrant oncogene-dependent proliferative signaling (Collado and Serrano, 2006, 2010; Campisi and d’Adda di Fagagna, 2007).

Several laboratories demonstrated that PML is a key regulator of oncogene-induced cellular senescence. PML achieves this effect through several complementary and interconnected mechanisms. Oncogenic K-RAS induces senescence in a p53-dependent manner. In this context, PML is transcriptionally upregulated and promotes the recruitment to PML-NBs of the p300/CBP transcriptional co-activator, which in turn leads to p53 acetylation and consequent transcriptional activation (Ferbeyre et al., 2000; Pearson et al., 2000). Notably, PML itself is a p53 target gene (de Stanchina et al., 2004). These observations led to the conclusion that oncogenic K-RAS induces cellular senescence through a feed-forward transcriptional mechanism that depends on the p53 tumor suppressor (de Stanchina et al., 2004).

Subsequently, it was also found that PML also induces senescence *via* the retinoblastoma (Rb) pathway in human fibroblasts (Mallette et al., 2004).

More recently, we showed that oncogenic K-RAS induces PML upregulation even in p53 null cells. Under these conditions, the PML 5′ untranslated mRNA region (5′ UTR) mediates selective uploading of the PML mRNA onto polyribosomes and its selective translation, leading to p53 independent PML upregulation. These data suggest that selective protein translation plays an important role in mediating an efficient oncogene-induced replicative senescence response (Scaglioni et al., 2012).

### Apoptosis

It is well established that PML promotes apoptosis. For instance, *Pml* null thymocytes show a reduction of DNA damage-induced apoptosis when compared to their wild type counterparts (Wang et al., 1998b). This is, at least in part, due to the ability of PML to physically interact with p53 regulating its transcriptional activity. These findings led to the conclusion that inactivation of PML impairs the ability of p53 to induce expression of specific pro-apoptotic and growth-inhibiting target genes such as *p21* and *Bax*. Furthermore, PML is required for Fas and caspase-dependent apoptotic activity (Wang et al., 1998b; Guo et al., 2000).

Another function of PML in apoptosis has been associated to the PML isoforms that are located in the cytoplasm. PML accumulates in PML-NBs, however some PML isoforms have been described in the cytoplasm (Lin et al., 2004). It was reported that cytoplasmic PML aggregates in a macromolecular complex with the 1,4,5-triphosphate receptor (IP_3_R), AKT, and the protein phosphatase 2a (PP2a) at mitochondria-associated membranes (MAMs). Here PML regulates IP_3_R-mediated Ca^2+^ release from the endoplasmic reticulum with the consequent induction of apoptosis (Giorgi et al., 2010).

### Neoangiogenesis

Promyelocytic leukemia tumor suppressor gene deficiency increases neoangiogenesis in mouse models and its deficiency correlates with increased vessel density in human cancers. Indeed, PML deficiency increases pro-angiogenic factors such as vascular endothelial growth factor (VEGF) and hypoxia-inducible factor 1 (HIF1). PML achieves this effect by repressing HIF1 transcriptional activity, and controlling HIF1 accumulation by the negative regulation of the AKT-mTOR pathway (Bernardi et al., 2006).

### Cell migration

Promyelocytic leukemia tumor suppressor gene can negatively regulate the expression of the β1 integrins. Therefore, PML is involved in the regulation of cell migration preventing metastasis (Reineke et al., 2010).

### DNA damage response

Even though the mechanisms are still not completely clear, PML has been shown to be involved in the regulation of the response to DNA damage.

Promyelocytic leukemia tumor suppressor gene-nuclear bodies have been proposed as highly dynamic structures in which several DNA repair factors transit in order to be redistributed to DNA damage foci. Accordingly, PML-NBs have been functionally associated to several pathways regulating DNA repair, including non-homologous end-joining repair (NHEJ), homologous recombination repair (HR), as well as the alternative lengthening of telomerases (ALT) (Dellaire and Bazett-Jones, 2004).

### Antiviral defense

Several viral proteins interact with PML and PML-NBs (Everett, 2001). Numerous studies underline the role of PML and PML-NBs in host antiviral response. For instance, interferon (IFN) stimulation leads to PML upregulation promoting PML-NBs formation (Chelbi-Alix et al., 1995; Regad and Chelbi-Alix, 2001; Geoffroy and Chelbi-Alix, 2011). Finally, several DNA and RNA viruses, such as herpes viruses and rabies viruses, encode proteins that co-localize with PML disorganizing the PML-NBs. This observation suggests that viruses disrupt PML-NBs as a strategy to evade cellular resistance mechanism to viral infections (Geoffroy and Chelbi-Alix, 2011).

### Hematopoietic stem cell maintenance

Asymmetric stem cell division is thought to be critical to ensure self-renewal of the stem cells reservoir. Through this process stem cells give rise to two daughter cells with different cellular fates: one daughter cells will maintain the stem cell pool, while the second daughter cells is programed to undergo differentiation. PML has been recently involved in the maintenance of HSCs (Ito et al., 2008) and in the regulation of asymmetric division of stem cells. PML achieves this effect by regulating the Peroxisome Proliferator-Activated Receptors (PPAR)-δ, which in turn promotes fatty acid oxidation (FAO). In this setting, inactivation of PML, PPAR-δ, or FAO results in symmetric HSCs division and loss of their self-renewal capacities (Ito et al., 2012).

## Post-Translational Modification of PML

Several post-translational modifications regulate PML activity. These modifications are described below.

### Phosphorylation

Promyelocytic leukemia tumor suppressor gene is phosphorylated at serine and tyrosine residues. For instance, the mitogen-activated protein kinase Extracellular signal Regulated Kinases 2 (ERK2) has been shown to phosphorylate PML on several residues. These modifications have been implicated in the degradation of PML upon treatment with arsenic trioxide (ATO) (Hayakawa and Privalsky, 2004). Ataxia-Telangiectasia and RAD3-Related (ATR) and Checkpoint Kinase 2 (CHK2) also phosphorylate PML in a DNA damage-dependent way (Bernardi et al., 2004; Yang et al., 2006). Moreover, phosphorylation by Casein Kinase 2 (CK2) induces PML degradation in a proteasomal/ubiquitin-dependent way (Scaglioni et al., 2006).

### SUMOylation

SUMOylation is one of the most intensely studied post-translational modifications of PML and of PML-RARA. Both SUMO1 and SUMO2/3 covalently bind PML (Kamitani et al., 1998a,b; Fu et al., 2005); in addition, PML binds to the SUMO E2-ligases UBC9 (Borden, 2002). SUMOylation of PML and PML-RARA have been associated to seemingly opposed activities. For example, it was reported that SUMOylation of PML facilitates the assembly of the PML-NBs promoting tumor suppressive responses and that SUMOylation of PML-RARA is essential for its leukemogenic activities (Zhong et al., 2000b; Zhu et al., 2005; Shen et al., 2006), however other reports indicated that, in cells exposed to ATO, SUMOylation of PML and PML-RARA promote their ubiquitin-mediated degradation (Lallemand-Breitenbach et al., 2008; Tatham et al., 2008). More recently, we demonstrated that the Protein Inhibitor of Activated STAT 1 (PIAS1) SUMO E3-ligase SUMOylates PML and its oncogenic counterpart PML-RARA. This process leads to the ubiquitin-mediated degradation of both PML and PML-RARA (Rabellino et al., 2012). Taken together, these studies have led to the working hypothesis that poly SUMOylation represents a molecular switch that triggers the ubiquitin-mediated degradation of both PML and PML-RARA (de The and Chen, 2010; de The et al., 2012). We will discuss later in this review the critical data that support this hypothesis.

### Acetylation

Another PML post-translational modification is acetylation. PML acetylation on the lysines 487 and 515 has been proposed to be associated with the increase of PML SUMOylation, suggesting that the acetylated form of PML is preferentially SUMOylated. PML acetylation is enhanced by the Histone deacetylase (HDAC) inhibitor trichostatin A (TSA), suggesting a role of PML acetylation in TSA-induced apoptosis (Hayakawa et al., 2008).

### Ubiquitination

Finally, PML can be also ubiquitinated. PML ubiquitination plays an essential role in the regulation of PML functions and activity. Importantly, aberrant PML ubiquitination and degradation has been reported to occur in various types of human tumors. For these reasons, PML ubiquitination and degradation will be discussed in greater detail later in this review.

## PML Degradation

Promyelocytic leukemia tumor suppressor gene plays a pivotal role in tumor suppression, apoptosis, and senescence, and in the control of viral infections. For these reasons, PML degradation would provide a selective advantage in tumor initiation and progression or a strategy used by viruses to evade the antiviral cellular mechanisms.

The first evidence of the disruption of PML activity came from the study of PML-RARA in APL, where PML tumor suppressive functions are disrupted due to the fusion with the RARA (Rego et al., 2001). In addition, complete or partial loss of PML protein expression has been observed in multiple human cancer types, such as prostate and colon adenocarcinoma, breast carcinoma, lymphoma, CNS tumors, and germ cell tumors (Koken et al., 1995; Gambacorta et al., 1996; Gurrieri et al., 2004a; Chen et al., 2012).

The PML gene rarely undergoes somatic mutations in tumors other than APL and its promoter does not undergo epigenetic silencing (Gurrieri et al., 2004a). Instead, deregulated ubiquitination appears to be the common mechanism accounting for PML loss in cancer. Several mechanisms have been reported to be involved in PML ubiquitination (Fanelli et al., 2004; Scaglioni et al., 2006; Lallemand-Breitenbach et al., 2008; Louria-Hayon et al., 2009; Reineke et al., 2010; Lim et al., 2011; Yuan et al., 2011; Chen et al., 2012; Rabellino et al., 2012; Wolyniec et al., 2012). We will review their biological significance in tumorigenesis and in viral infection.

### PML-RARA degradation

Historically, the first evidence of PML degradation was obtained in APL leukemic blasts treated with ATO. The *t*(15;17) of APL is a reciprocal and balanced translocation giving rise to both PML-RARA and a RARA-PML fusion proteins. As a result of this translocation, both PML and RARA are reduced to hemizygosity. Extensive work from several laboratories has characterized the properties and mechanisms of action of PML-RARA (Scaglioni and Pandolfi, 2007; Ablain and de The, 2011).

In this disease, treatment with ATO leads to complete APL remission. Molecular studies demonstrated that ATO treatment leads to ubiquitin-proteasomal degradation of PML-RARA leading to the conclusion that this property of ATO is responsible for its therapeutic effect (Wang and Chen, 2008; de The and Chen, 2010; de The et al., 2012).

Arsenic trioxide induces PML-RARA ubiquitination and consequent proteasomal degradation through a mechanism that involves the SUMOylation of its PML moiety. This process has been recently unveiled in detail. Mechanistically, ATO binds directly the RING domain of the PML moiety of PML-RARA, facilitating its oligomerization and SUMOylation (Zhang et al., 2010); moreover, ATO induces the production of reactive oxygen species that allow the formation of PML-RARA-multimers, which aggregate into PML-NBs, the cellular compartment where their degradation ultimately occurs (Lallemand-Breitenbach et al., 2001, 2008; Tatham et al., 2008; Jeanne et al., 2010; Rabellino et al., 2012).

It was reported that SUMOylation at lysine 160 of the PML moiety is essential for the proteasome-targeting of PML-RARA (Lallemand-Breitenbach et al., 2001), however the SUMOylation of other lysines may be involved in the process as well (Rabellino and Scaglioni, unpublished data). The groups of R. Hay and H. de Thé clarified the molecular mechanism that allows the degradation of SUMOylated PML and PML-RARA. In cells treated with ATO the ubiquitin E3-ligase Ring-Finger protein 4 (RNF4) is rapidly recruited into the PML-NBs where it recognizes poly-SUMOylated chains covalently bound to PML and PML-RARA, leading to their ubiquitination and consequent degradation (Lallemand-Breitenbach et al., 2008; Tatham et al., 2008). RNF4 is a member of the SIM/RING-finger family. These proteins can directly couple the ubiquitin machinery to poly-SUMOylated substrates through their SIM domain. Due to this peculiar characteristic, this family of protein is indicated as SUMO Targeted Ubiquitin Ligases (STUBbLs) (Sun et al., 2007).

The SUMO E3-ligase that mediates the SUMOylation of PML and PML-RARA has been elusive until recently. We showed that PIAS1 is a PML and PML-RARA SUMO E3-ligase. Ectopic expression of PIAS1 increases PML-RARA SUMOylation and strikingly increases the ability of ATO to degrade PML-RARA. Knock down of PIAS1 in NB4 cells, an APL cell line that recapitulates the effects elicited by ATO in primary APL cells (Roussel and Lanotte, 2001), significantly reduces the ability of ATO to degrade PML-RARA causing a significant resistance to ATO-induced apoptosis (Rabellino et al., 2012). It should be noted that PIAS1 mediates also the degradation of PML in cells treated with ATO, however this event is apparently inconsequential in APL. This is probably due to the fact that PML-RARA is the dominant driving force of APL (Scaglioni and Pandolfi, 2007; de The and Chen, 2010).

**Figure 2:**
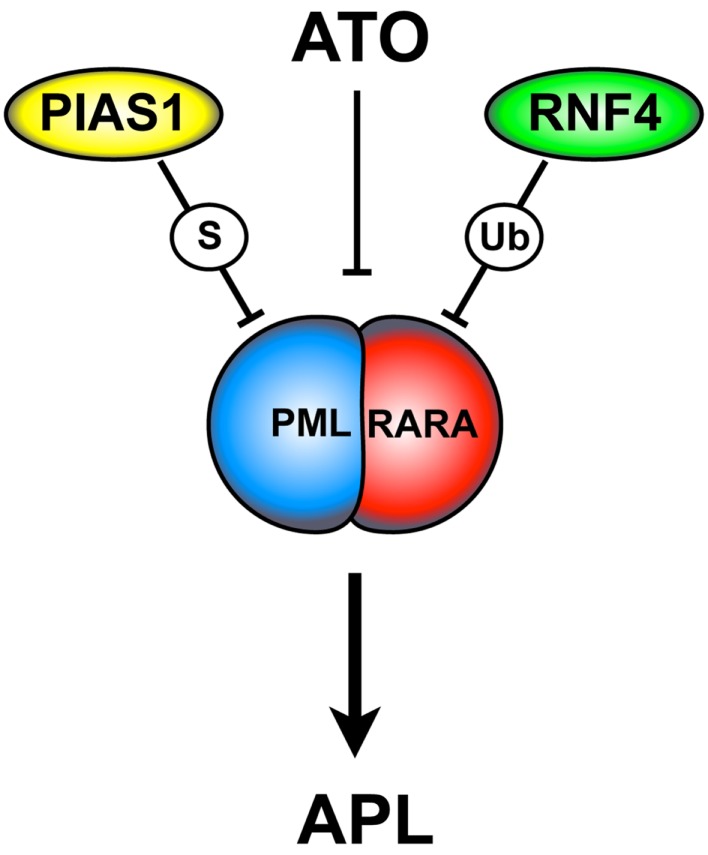
**ATO-dependent PML-RARA SUMOylation and degradation**. ATO triggers PML-RARA degradation leading to APL remission. This process requires the PIAS1 SUMO E3-ligase and the ubiquitin E3-ligase RNF4. ATO is thought to directly bind to the PML moiety of PML-RARA. This event causes a conformational change that allows PML-RARA poly-SUMOylation and its interaction with RNF4. ATO, arsenic trioxide; S, SUMOylation; Ub, ubiquitination.

Collectively, these findings elegantly describe the molecular mechanisms involved in PML-RARA degradation (Figure 2), rising the opportunity to improve APL therapy.

Even though the molecular mechanism of ATO-dependent PML-RARA degradation has been described in great detail, several questions are still open. For example, we demonstrated that ATO stimulates PIAS1 activity, increasing its ability to SUMOylate PML-RARA (Rabellino et al., 2012). However, the mechanism of activation of PIAS1 is still unknown, and whether ATO triggers PIAS1 activity through direct binding to its RING domain (as is the case for RNF4) or through other mechanisms is still unknown.

Furthermore, the model described above is at first sight at odds with the longstanding observation that SUMOylation of PML-RARA at lysine 160 is required for APL leukemogenesis (Zhu et al., 2005). This observation is consistent with our finding that, in the absence of ATO, PIAS1-mediated SUMOylation up-regulates PML-RARA and is not sufficient to induce its degradation (Rabellino et al., 2012). However ATO dramatically enhances PML-RARA poly-SUMOylation. Thus, it is possible that ATO maximally activates PIAS1 or that it promotes the recruitment of yet to be identified proteins that enhance the processivity of PIAS1 allowing the synthesis of longer SUMO chains. With unpublished experiments we discovered that in the presence of ATO PIAS1 SUMOylates sites in the PML moiety other than K160, but whether or not these residues have a biological role in determining the response to ATO treatment is not yet known. These questions will keep the aficionados of this field busy in the future.

### CK2-dependent PML degradation

The group of Pandolfi was the first to report an extensive analysis of PML status in human cancer. These investigators determined that PML is commonly lost in several cancers types. However, this group did not detect somatic mutations or promoter methylation events that justified PML deficiency (Gurrieri et al., 2004a,b). This analysis has been confirmed with large mutational studies in human cancer (for example the Sanger Cosmic database). Subsequently, it was reported that PML is degraded in immortalized and tumor-derived cell lines through a mechanisms that involves proteasomal dependent ubiquitin-mediated degradation. Mutational analysis of PML revealed the presence of a C-terminal phosphodegron essential for PML ubiquitination (Scaglioni et al., 2006).

Casein Kinase 2 is a highly conserved and ubiquitously expressed serine/threonine kinase (Pinna, 2002). CK2 is frequently activated in a wide range of human cancers and its expression in transgenic mice can induce mammary tumor formation and lymphomas (Seldin and Leder, 1995; Landesman-Bollag et al., 2001).

Casein Kinase 2 promotes direct phosphorylation of PML on the serine 517 (note that this nomenclature applies to PML isoform IV lacking exon 5) located in the PML degron, leading to PML ubiquitin-mediated degradation. Accordingly, PML mutants that cannot be phosphorylated by CK2 show a resistance to ubiquitin-mediated degradation and increased tumor suppressive functions. In this process, the activity of the p38 MAPK is required in order to activate CK2, and pharmacological inhibition of CK2 enhances the PML tumor suppressive activity *in vivo*. Finally, it has been shown an inverse correlation between the level of PML proteins and CK2 kinase activity in human lung cancer specimens (Scaglioni et al., 2006). These observations lead to the hypothesis that the phosphorylation of the serine 517 on the PML degron by CK2 is required for the interaction with a specific ubiquitin E3-ligase, but its identity is still unknown. In conclusion, since it has been shown that *CK2* gene amplification is a marker of poor prognosis in NSCLC (O-charoenrat et al., 2004), a therapy that specifically inhibits CK2 may be particularly effective by inducing restoration of PML and its tumor suppressive functions. Notably, several specific CK2 inhibitors have demonstrated antitumor activity in preclinical trials. Moreover, a CX-4945, a specific pharmacologic inhibitor of CK2, is being tested in early clinical trials in cancer patients: these drugs could be instrumental in restoring PML in cancer cells (Hanif et al., 2010; Siddiqui-Jain et al., 2010; Ferguson et al., 2011; Sarno et al., 2011; Cozza et al., 2012).

This work described how oncogenic stress through the activation of CK2 is able to induce PML degradation in cancer cells both *in vitro* and *in vivo*. However, some links are missing from this working model. For example, the ubiquitin E3-ligase involved in the PML ubiquitination is still unknown. Moreover, the initial report, did not determine whether SUMOylation, which has been involved in PML degradation in cells exposed to ATO, is involved in CK2 mediated PML degradation.

### PIAS1-dependent PML degradation

SUMOylation has been implicated in the regulation of both PML and PML-RARA (Lallemand-Breitenbach et al., 2001, 2008; Tatham et al., 2008). Moreover, SUMOylation has been implicated in PML-NBs formation (Zhong et al., 2000b; Shen et al., 2006; Bernardi and Pandolfi, 2007) and in regulation of PML-mediated apoptosis (Hayakawa and Privalsky, 2004). These observations indicate that SUMOylation plays an important role in PML regulation. We hypothesized that the discovery of the SUMO E3-ligase that mediates PML SUMOylation would reveal functional and mechanistic insights onto the regulation of PML biological properties.

**Figure 3:**
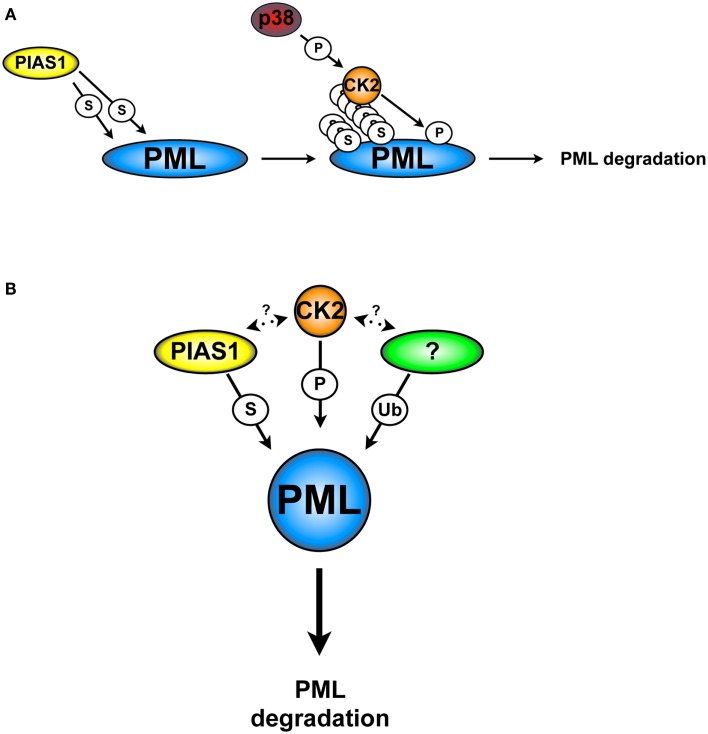
**CK2 and PIAS1-dependent PML degradation**. **(A)** PIAS1-dependent PML SUMOylation is required for the phosphorylation of the PML degron by CK2 and subsequently degradation. **(B)** PIAS1, together with CK2 and an unknown ubiquitin E3-ligase cooperate in order to lead to PML degradation. S, SUMOylation; Ub, ubiquitination; P, phosphorylation. Arrows indicate possible cross-talks.

We tackled this question by identifying PML interacting proteins with the yeast two-hybrid screening. We found that PIAS1 and PIASxα interact with PML, promoting its SUMOylation. Surprisingly, we found that only PIAS1-dependent PML SUMOylation leads to PML ubiquitination and degradation; on the contrary, PIASxα leads to PML stabilization. Consistent with these results, PIAS1 silencing in a panel of NSCLC cell lines up-regulates PML protein levels and leads to a significant PML-dependent anti-proliferative effects. In this context, its notable that PIAS1-dependent SUMOylation increases the interaction between CK2 and PML, which in turns promotes PML phosphorylation at serine 517 and degradation. Based on these findings, we propose a model in which PIAS1-dependent PML SUMOylation is required for the CK2/PML interaction and phosphorylation of the PML degron (Figure 3A).

Finally, we found that PIAS1 and PML expression are inversely correlated in NSCLC cell lines and in primary lung cancers (Rabellino et al., 2012) and prostate (Rabellino and Scaglioni, unpublished data). Consistently with these studies, we also found that the *PIAS1* gene is amplified in a subset of NSCLC cell lines (Rabellino et al., 2012). These data indicate that PIAS1 promotes tumorigenesis through downregulation of PML.

The SUMO E3-ligases PIASs have been mainly implicated in the regulation of innate immunity through epigenetic mechanisms (Liu et al., 2004, 2010; Shuai and Liu, 2005; Rytinki et al., 2009). Moreover, it has been suggested that PIAS1 may regulate oncogenic networks through its ability to inhibit tumor suppressors such as p53, BRCA1, and STAT1 (Schmidt and Muller, 2002; Galanty et al., 2009; Wu and Chiang, 2009). Therefore, our work reveled a novel function of PIAS1.

Taken together, these findings show an elaborated cellular network, in which SUMOylation, CK2, and a not yet identified ubiquitin E3-ligase cooperate in order to trigger PML degradation (Figure 3B). For instance, is tempting to speculate that any of the PML ubiquitin E3-ligase that have been described so far may play a role in this process. However, there are no reports in the literature that support this hypothesis. Finally, the identification of specific inhibitors able to target PIAS1 activity may provide a strategic alternative to inhibit PML degradation.

The finding that PML, PIAS1, and CK2 physically interact suggests that PIAS1 and CK2 may cross talk. This hypothesis is supported by the observation that CK2 phosphorylates PIAS1 *in vitro* (Stehmeier and Muller, 2009). Hence, future studies should determine whether CK2 phosphorylates PIAS1 also in cells and whether this event has any functional relevance.

It also remains to be determined whether other members of the PIAS family are PML SUMO E3-ligases and whether they regulate any of the activities ascribed to PML. Furthermore, the ubiquitin E3-ligase(s) involved in the final process of PML ubiquitination and degradation in cancer cells is still unknown and future investigations should be directed to its/their identification. Finally, it is not clear yet if the ubiquitination occurs on PML itself, or on the SUMO chains as described for PML-RARA (Lallemand-Breitenbach et al., 2008; Tatham et al., 2008).

### Hypoxia-dependent PML ubiquitination

The ability to induce neoangiogenesis is one of the hallmarks of cancer (Hanahan and Weinberg, 2011), and the transcription factor HIF1 is a key regulator of the adaption of cancer cells to conditions of hypoxia (Semenza, 2010).

The protein complex KLHL20-Cul3-ROC1 is an ubiquitin E3-ligase involved in the regulation of HIF signaling through the poly-ubiquitination and degradation of the Death-associated Protein Kinase (DAPK), a mediator of IFN-induced cell death. The KLHL20-dependent DAPK ubiquitination is suppressed in cells stimulated with IFN, which induce the KLHL20 sequestration into the PML-NBs (Lee et al., 2010).

It was recently reported that KLHL20 cooperates with the Cyclin-dependent Kinase 1/2 (CDK1/2) and the Peptidyl-prolyl cis/trans isomerase Pin1 to mediate PML degradation in hypoxic conditions. Under hypoxia, HIF1 induces the transcription of KLHL20, increasing its accumulation into PML-NBs. In this scenario, two different post-translational modifications of PML make possible its interaction with KLHL20: first, PML isoform I (PML-I) is phosphorylated on serine 518 by CDK1 and CDK2. Second, phosphorylated PML-I undergoes Pin1-dependent prolyl-isomerization. Notably, the authors reported that hypoxia-dependent PML degradation, not only attenuates PML tumor suppressor activity, but also participates to a feedback mechanism that maximizes the production of HIF1 during hypoxic stress. These data are consistent with the observation that in human prostate cancers PML inversely correlates with the over expression of HIF1, KLHL20, and Pin1 (Yuan et al., 2011).

These data led to the conclusion that the KLH20-Cul3 complex is an ubiquitin E3-ligase for PML. However, the authors found that in this case, SUMOylation is dispensable for PML ubiquitin-mediated degradation. Therefore, it is not clear whether the KLH20-Cul3 complex is mainly responsible for PML degradation in conditions of hypoxia or whether it plays a significant role also in regulating the role of PML in other conditions of oncogenic stress. Moreover, it remains to be determined in which tumor types this phenomenon may occur. Thus, future experiments will be needed to determine the significance of the KLH20-Cul3 complex to PML degradation *in vivo*. We reason that these studies will benefit from studies with genetically engineered mouse models to determine the biological significance of these phenomena *in vivo*.

### SIAH1/2-dependent PML and PML-RARA degradation

SIAH proteins are RING-finger-containing ubiquitin E3-ligases involved in the degradation of transcriptional regulators, components of the cell cycle machinery, and proteins involved in tumorigenesis (House et al., 2009; Lipkowitz and Weissman, 2011). The mammalian SIAH1 and SIAH2 have been reported to interact with PML and PML-RARA inducing their degradation. More in detail, SIAH1 and SIAH2 interact with the RING domain of PML and PML-RARA triggering their proteasome-mediated degradation. Accordingly, overexpression of SIAH1 and SIAH2 in APL cell lines, partially restore PML-RARA-induced differentiation block in APL blast through the degradation of PML-RARA (Fanelli et al., 2004).

The conclusions of this study are limited by the fact that these data were obtained with proteins ectopically expressed in cell lines cultured *in vitro*. Moreover, it is noteworthy that overexpression of SIAH1 and SIAH2 in PML-RARA expressing cells only partially rescues cells differentiation, raising the possibility that PML-RARA is not a physiological substrate of SIAH1 and SIAH2. Studies with genetically engineered mice would be needed to test the role of SIAH1 and SIAH2 in APL.

### ERK2/Pin1-dependent PML degradation

Pin1 is over expressed in a wide range of human tumors (Wulf et al., 2005). Due to its ability to interact with different proteins families, Pin1 has been found to be able to affect phosphorylation status, protein-protein interactions, cellular localization, and protein stability (Galat, 2003). It was reported that in breast cancer cell lines Pin1 binds PML, inducing its downregulation. In this scenario, phosphorylation of PML on two key serine residues (serine 403 and serine 505) by the kinase ERK2 facilitates the recruitment of Pin1. In this study, inhibition of ERK2 with specific drugs or siRNA decreased the interaction between PML and Pin1, leading to upregulation of PML and PML-NBs formation. Conversely, stimulation of ERK2 with growth factor such as Epidermal Growth Factor (EGF) increases phosphorylation of PML and its downregulation (Reineke et al., 2008; Lim et al., 2011).

The MAP-kinase ERK2 is widely involved in eukaryotic signal transduction (Roskoski, 2012). Several extracellular signaling can activates the MAP-kinase cascade, including aberrant signaling from oncogenic stress or stimuli. From this point of view, a better understanding of the mechanisms involved in ERK2/Pin1-dependent PML degradation may provide new insight in the development of specific drugs targeted to prevent PML degradation.

This study is however limited by the fact that it was performed in a limited set of cultured breast cancer cell lines. Moreover, this study did not present any data obtained in genetically defined mouse cancer models or in primary human specimens. Furthermore, it remains to be addressed whether the CDK1/2-Pin1 and the ERK2/Pin1 mechanisms to ubiquitinate PML are complementary, mutually exclusive, or tumor type specific. Thus, future studies will need to determine whether these data are relevant in naturally occurring tumors. Should this be the case, it is noteworthy that several chemical inhibitors of MEK1 (the upstream regulator of ERK2) exist; thus, this strategy could be tested in clinic.

### E6AP-mediated PML ubiquitination

E6AP was the first mammalian ubiquitin E3-ligase to be identified. Initially, E6AP was identified as the ubiquitin E3-ligase that cooperates with the human papillomavirus (HPV) protein E6 to promote p53 degradation (Talis et al., 1998). E6AP interacts with PML, partially residing into the PML-NBs. It was reported that E6AP overexpression leads to PML downregulation in an proteasome/ubiquitin-dependent way; accordingly E6AP null cells show higher expression of PML proteins (Louria-Hayon et al., 2009).

DNA damage triggers accumulation of PML and increases PML-NBs formation (Bernardi and Pandolfi, 2007). Notably, E6AP deficient cells show an increase of DNA damage-dependent apoptosis, due to an accumulation of PML into to the nucleus with a consequent increase of PML-dependent response. Finally, E6AP is also involved in ATO-dependent PML degradation (Louria-Hayon et al., 2009).

The role of E6AP in PML degradation has also been investigated in B-cell lymphomagenesis. Partial loss of E6AP attenuates MYC-induced B-cell lymphomagenesis. In this model, tumor suppression is achieved by the induction of cellular senescence but not apoptosis. Accordingly, partial loss of E6AP leads to PML restoration and subsequently induction of PML-dependent senescence. Indeed, B-cell lymphomas lacking EA6P express elevated level of PML and PML-NBs, with a concomitant increase of markers of senescence. Accordingly, E6AP expression levels are elevated in 43% of human Burkitt lymphoma derived cells lines and in 60% of primary human Burkitt lymphomas (Wolyniec et al., 2012).

Taken together, these data indicate an important role of E6AP in PML degradation. Noteworthy, E6AP may contribute to ATO-induced PML degradation, and further analysis should be done to understand if E6AP and RNF4 interact in the promotion of PML degradation or whether they participate in distinct processes.

Promyelocytic leukemia tumor suppressor gene protein expression is frequently lost in non-Hodgkin lymphomas (Gurrieri et al., 2004a) and the expression of EA6P is elevated in about 60% of human Burkitt’s lymphoma. The model provided gives a molecular explanation of the down-regulation of PML in non-Hodgkin lymphomas, suggesting a direct connection between E6AP expression and PML degradation in this disease. Importantly, these data are supported by a correlation with *in vivo* model and human specimens, providing confidence in their relevance *in vivo*. Finally, these findings strongly suggest that restoration of PML expression in non-Hodgkin lymphomas may provide an attractive therapeutic approach.

### Virus-dependent PML degradation

Viral infections often result in PML-NBs disruption and PML degradation (Everett, 2001).

Historically, the first evidence of the interaction between viruses and PML came from the observation that infection of cells with Herpes virus 1 (HSV-1) led to the rapid disruption of the PML-NBs (Maul et al., 1993). Further analysis confirmed that the HSV-1 regulatory protein ICP0 is sufficient to induce PML degradation in a proteasome-dependent way (Everett and Maul, 1994; Chelbi-Alix and de The, 1999). Similar to HSV-1, infection with human Cytomegalovirus (HCMV) causes PML-NBs disruption: in this process, the viral protein IE1 is necessary and sufficient to promote this effect. However in this case, IE1 is not involved in PML degradation, but in its chromatin redistribution (Ahn et al., 1998).

Another example of viral protein able to disrupt the PML-NBs is BZLF1, from the Epstein–Barr virus (EBV). In this case, expression of BZLF1 alone is sufficient to induce PML-NBs disruption and the loss of the SUMOylated forms of PML. Interestingly, BZLF1 can be SUMOylated, and it has been reported that BZLF1 competes with PML for its SUMOylation. This observation suggests that if a cellular component of the SUMOylation machinery is limiting, BZLF1 could inhibit PML SUMOylation by the direct competition with the limiting factor (Adamson and Kenney, 2001).

Taken together, these data suggest that the disruption of PML-NBs and the degradation of PML are an important step for the lytic cycle of all herpes virus (Adamson and Kenney, 2001; Everett, 2001).

Other viral infections have also been association with the disruption of PML-NBs (Everett, 2001; Geoffroy and Chelbi-Alix, 2011). For example, it has been suggested that HIV1 infection triggers delocalization of PML into the cytoplasm, however, other groups have found that HIV1 does not modify PML-NBs (Turelli et al., 2001; Geoffroy and Chelbi-Alix, 2011). Therefore, the role of HIV in the regulation of PML-NBs and PML is still unsettled.

The antiviral activity of PML resides on its ability to re-organize the PML-NBs in order to activate an efficient antiviral response. For this reason, it has been suggested that the ability of certain virus to interact with the PML-NBs formation inducing PML down-regulation is a strategy to evade the antiviral effect of PML (Geoffroy and Chelbi-Alix, 2011). Thus, a better understanding of the molecular mechanisms involved in virus-dependent PML degradation may provide new insight in a more efficient antiviral therapies or strategies to prevent viral infections. Given the advances in our understanding of the mechanism that lead to PML ubiquitin-mediated degradation in cancer cells, future studies will undoubtedly test whether viruses utilize the same cellular machinery that cancer cells use.

## Conclusion

**Figure 4:**
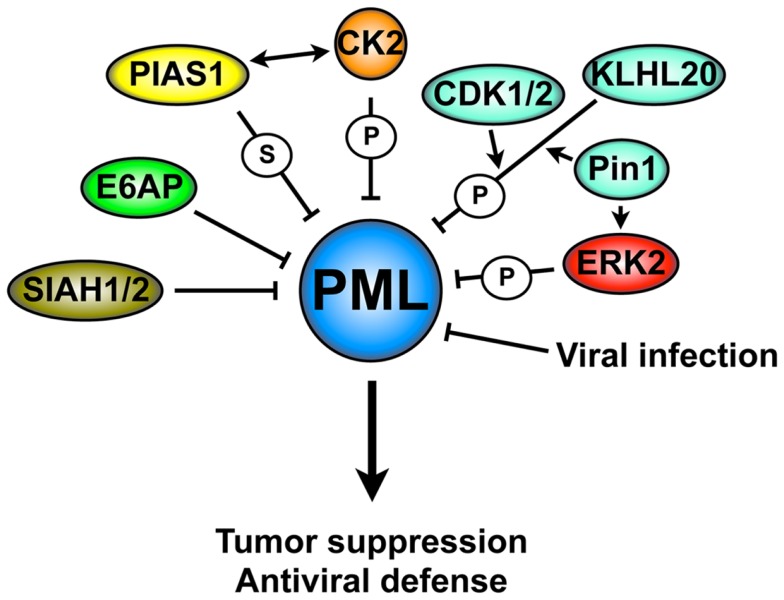
**Mechanisms of PML degradation**. A complex molecular network is involved in PML degradation: these mechanisms include several post-translational modifications that trigger PML ubiquitination and degradation, resulting in the loss of its tumor suppressive activity or antiviral properties. S, SUMOylation; P, phosphorylation.

Promyelocytic leukemia tumor suppressor gene is a key tumor suppressor and its inactivation through aberrant degradation has been found in several human cancers types. Moreover, PML downregulation plays an important role in promoting viral infections. A large body of literature indicates that PML is rarely mutated and its downregulation occurs predominately at the post-translational level. Several pathways have been reported to trigger PML ubiquitination and degradation (Figure 4). This situation is reminiscent of the degradation of other tumor suppressors, which is often achieved through multiple context-dependent networks. It will be important in the future to determine which mechanisms are the critical regulators on PML degradation in specific cancers *in vivo*. We reason that these studies will most likely benefit from the availability of faithful and genetically defined mouse cancer models and correlative studies in primary human samples. We are looking forward to these developments with the expectation that pharmacologic targeting of the networks that control PML degradation will lead to specific therapies for cancer and for several viral infections.

## Conflict of Interest Statement

The authors declare that the research was conducted in the absence of any commercial or financial relationships that could be construed as a potential conflict of interest.
